# Hard Cancre of Conjunctiva

**Published:** 1916-10

**Authors:** 


**Hard Cancre of Conjunctiva.** Henri Fromaget of Boulogne-sur-Mer, Jour. de Med. de Bordeaux, reports a case in a boy of ten, involving the bulbar conjunctiva but not the cornea, without explanation, painless, cured by novarsenobenzol, the doses increasing from 15 to 60 centigrams, from Jan. 7 to Feb. 11 six altogether.

